# *Dermacentor reticulatus* and *Babesia canis* in Bavaria (Germany)—A Georeferenced Field Study with Digital Habitat Characterization

**DOI:** 10.3390/pathogens9070541

**Published:** 2020-07-07

**Authors:** Cornelia Silaghi, Lisa Weis, Kurt Pfister

**Affiliations:** 1 Comparative Tropical Medicine and Parasitology, Faculty of Veterinary Medicine, Ludwig-Maximilians-Universität München, 80802 Munich, Germany; mail@lisaweis.de (L.W.); kpfister@duc.ch (K.P.); 2 Institute of Infectology, Friedrich-Loeffler-Institut, Greifswald Isle of Riems, 17493 Greifswald, Germany

**Keywords:** *Dermacentor reticulatus*, *Canine babesiosis*, georeferenced, ecotones, habitat characterization, red deer

## Abstract

The hard tick *Dermacentor reticulatus* transmits *Babesia canis*, the causative agent of canine babesiosis. Both the occurrence and local distribution of *D. reticulatus* as well as infection rates of questing ticks with *B. canis* are thus far poorly known in Bavaria, Germany. The objectives of this study were to conduct (1) a georeferenced field study on the occurrence of *D. reticulatus* with digital habitat characterization and (2) a PCR analysis of *D. reticulatus* collected in Bavaria for infection with *B. canis*. *Dermacentor reticulatus* were collected by flagging at 60 sites specifically selected according to habitat conditions and screened individually for *Babesia* DNA. A digital habitat characterization for *D. reticulatus* was performed according to results of the field analysis including the parameters land use, proximity to water, “potential natural vegetation”, red deer corridors and climate data. Altogether, 339 *D. reticulatus* ticks (214 females and 125 males) were collected between 2010 and 2013 at 12 out of 60 sampling sites. All 12 sites were characterized by high humidity with marshy areas. *Babesia canis* DNA was detected in 1 out of 301 (0.3%) questing *D. reticulatus* in Bavaria. The digital habitat characterization revealed 15 forest areas in Bavaria with similar ecological characteristics as the sites positive for *D. reticulatus*.

## 1. Introduction

The hard tick *Dermacentor reticulatus* can transmit zoonotic pathogens such as *Rickettsia raoultii* and *Francisella tularensis* as well as the protozoon *Babesia canis*, the causative agent of canine babesiosis, a severe infectious disease in dogs [1,2]. Canine babesiosis is increasingly also reported as an autochthonous disease in Germany [1]. Possible reasons for such autochthonous infections include an expanding distribution of *D. reticulatus* due to changes of climate, land use and host density and distribution [1,3,4]. A similar pattern has been shown for the distribution of *Ixodes scapularis* and the incidence of Lyme disease caused by *Borrelia burgdorferi* sensu lato. An increase in temperature due to climate change led to an increase in the incidence of Lyme disease [5].

Hence, accurate and updated knowledge on the distribution of this tick species as well as its potential infections rates with *B. canis* is of utmost importance, particularly with regard to potentially necessary prevention measures.

First investigations on the distribution of *D. reticulatus* in Germany initially reported its absence in Germany [6], but since then, several *D. reticulatus* foci have been reported [7,8,9,10,11,12,13]. Furthermore, there are also some reports of *D. reticulatus* on dogs, roe deer and red deer [3,14,15,16].

*Dermacentor reticulatus* prefer rather humid habitats (e.g., alluvial forests and swamps) where they can survive flooding for certain periods of time [17]. Originally, *D. reticulatus* was reported only from areas with a lot of humidity, but there have also been reports from habitats with a drier character [7,9,18]. Since such conditions are largely present throughout Germany, further spreading of *D. reticulatus* is to be expected [19].

In Bavaria, a federal state in the southeast of Germany, *D. reticulatus* was first reported in the area of Regensburg and near Munich [20,21]. These findings raised the question of whether *D. reticulatus* has meanwhile also become endemic in Bavaria. 

To estimate the local infection risk for autochthonous canine babesiosis, prevalence rates must be determined in questing *D. reticulatus*. Until now, *B. canis* has only been detected in questing *D. reticulatus* in Germany in Saarland in western Germany [8]. Other detections were from engorged ticks collected on dogs [16], which, however, may reflect the infection status of the dog and not necessarily the infection rates of ticks. 

To update the knowledge on the occurrence of *D. reticulatus* in Bavaria, Germany, the objectives of this study were to establish (1) a field study on the occurrence of *D. reticulatus* including a digital habitat characterization using both analog and digital climate data and (2) a PCR analysis of all collected *D. reticulatus* for a possible infection with *B. canis*. 

## 2. Material and Methods

### 2.1. Field Study for Dermacentor reticulatus

**Figure 1:**
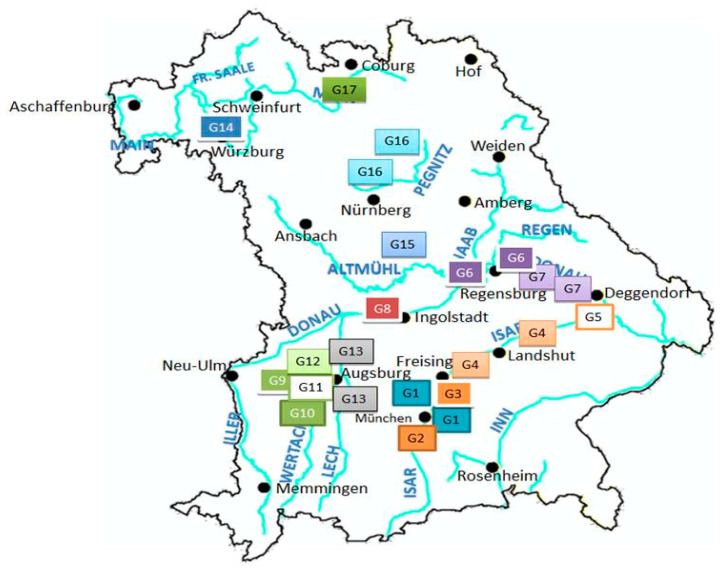
Location of the 17 sampling areas (including the 60 sampling sites shown in the supplementary table) in the federal state of Bavaria (Germany), sampled from 2010–2013.

The federal state of Bavaria (70,550 km^2^) in the southeast of Germany is located between 47°16′ and 50°34′ northern latitude and between 8°58′ and 13°50′ eastern longitude. The field study for collection of *D. reticulatus* was prepared by compiling a habitat list based on three different approaches with the aim of including areas with an increased probability of finding questing *D. reticulatus*: (i) habitats as described in historic and current scientific literature; (ii) geographic locations of previous descriptions and observations by veterinarians, pet owners and hunters; and (iii) Bavarian rivers with both natural and renatured alluvial forests (data from the Bavarian State Ministry of the Environment and Consumer Protection). Due to the large number of river areas, widely renatured areas containing large alluvial forests were chosen. According to these three search criteria, an initial habitat list was put together. Due to the vast number of sites, they were evaluated individually according to existing vegetation and terrain using the satellite maps from Google Maps of each region of interest (https://www.google.com/maps/). Following this, altogether 60 sampling sites were chosen, which were summed up in 17 sampling areas G1–G17 (Figure 1). All sites were sampled once yearly between March and June and again between August and November during the years 2010–2013, reflecting the two peak activity periods (spring and autumn) of *D. reticulatus*. In negative sites, *D. reticulatus* was assumed pseudoabsent. According to Wisz and Guisan (2009) [22], pseudoabsence describes the fact that absence of any species cannot definitely be proven. As far as tick sampling is concerned, the targeted tick species might be present in a sampling site, but might be temporarily unavailable to sampling efforts, e.g., due to climate conditions or any other reason. Positive sites were re-evaluated in one of the following activity periods. Sites which were positive in two consecutive activity periods were considered endemic foci.

Ticks were collected by the flagging method, and all ticks found on the flag were collected with tweezers and added to 15 mL Falcon tubes containing 70% ethanol and stored at room temperature. For each collection site, the coordinates and altitude were documented with a mobile navigation device (Garmin^®^ GPSmap 60CSx, Garmin, Garching, Germany). The temperature as well as the relative humidity were taken at a 50 cm height with a handheld thermohygrometer at the beginning of sampling (P33 Handmessgerät, Carl Roth GmbH, Karlsruhe, Germany). Additionally, the soil temperature at a 5 cm depth was taken with an insertion thermometer, also at the beginning of sampling. All collected ticks were identified in the laboratory under a stereomicroscope according to morphological criteria [23,24].

### 2.2. PCR Analysis for Babesia canis

All *D. reticulatus* ticks were washed twice in distilled water and air-dried, and DNA was extracted individually using the QIAamp DNA Mini Kit (Qiagen, Hilden, Germany) according to the manufacturer’s instructions for tissue. Ticks were disrupted in a TissueLyser (Qiagen) with 80 µL PBS and a 5 mm stainless steel bead in 2 mL Eppendorf tubes for 5 min at 20 bpm. Incubation was carried out overnight at 56 °C. Every 24 to 48 samples, a negative extraction control containing distilled and sterile water was included. The quality and quantity of the extracted DNA were checked with a photospectrometer (NanoDrop^®^ND-1000; PeqLab, Erlangen, Germany).

All DNA extracts were screened with a conventional PCR, targeting the *18S rRNA* gene as previously described [25,26]. In each PCR run, a negative control with molecular water and three positive controls (DNA of *B. canis*, *B. vogeli* and *B. divergens*, from naturally infected animals) were included.

PCR products were visualized with 2% agarose gel electrophoresis dyed with GelRed^TM^ (Fermentas Life Science, Leonrot, Germany) under UV light in a gel documenter (PeqLab). Positive samples were purified with a QIAquick PCR purification kit (Qiagen) and sent to an external laboratory for sequencing (Eurofins MWG Operon, Ebersberg, Germany). Sequences were evaluated with Chromas Lite (Technelysium) and compared with BLASTn with sequences previously deposited in GenBank. The 95% exact confidence interval was computed with Quickcalcs on www.graphpad.com.

### 2.3. Digital Habitat Analysis

All digital data were processed, if not stated otherwise, with ArcMap, version ArcGIS Desktop Education Edition 10 (ESRI^®^ Deutschland GmbH, Kranzberg, Germany). All digital datasets used were transformed into the coordinate system of the basic map (Geographic coordinate system of World Geodetic System: GCS_WGS_1984).

Data on the monthly and yearly average of temperature and rainfall for the whole area of Bavaria were obtained from Deutscher Wetterdienst (DWD, German weather service). The values obtained were the average values of 30 years (1971–2000). For each sampling area, climate data were determined in a buffer zone of 10 km (region of interest). The mean values of average monthly and yearly values were calculated for each region of interest. Similarly, the climate mean values of known *D. reticulatus* endemic areas determined in previous studies in Germany were also included: Upper Rhine, Schönbuch, Düben-Dahlen moor, Saarbrücken and Leipzig [7,8,9,20,27]. As the exact extent of distribution of *D. reticulatus* was not known in these previously described regions, the region of interest was determined to include described detection sites plus a buffer zone of 10 km. For the German-French border areas in the upper Rhine valley and Saarbrücken, data were available only for the German part of the area.

Digital data on land use and vegetation in dependency of soil type, available hosts and proximity to water bodies were also evaluated and calculated for the respective region of interest to characterize further suitable habitats for *D. reticulatus*.

For the analysis of land use, the digital data of the CORINE "Coordination of Information on the Environment" program of the European Environment Agency (EEA) was used. In this program, 44 land use classes are divided into 13 major classes. CORINE data are available as rectangular shapes of sides of about 150 × 150 km. Seventeen CORINE polygon maps were selected to cover the area of Bavaria. The maps were merged and cut to the shape of Bavaria, according to the state borders.

To analyze the proximity to water, the digital waterbody data, within which the water is classified into six waterbody types, of the digital official land survey register ALKIS^®^ of the Bavarian Surveying and Mapping Authority (BVV) were used. From the available data on waterbodies, river and lake data were used for further analysis. Vegetation complex in dependency of soil and terrain structure is shown in maps of the potential natural vegetation (PNV), which is provided by the Bavarian State Office for the Environment. The latter data set was analyzed only for the potential endemic habitat discovered in this study. Roe deer and wild boar are endemic in all areas of Bavaria, whereas red deer exist in defined geographic areas. Red deer move between habitats along old red deer corridors, which may also be used by other animals. Habitat maps of roe deer and red deer were available from the Bavarian State Office for the Environment, which were compiled according to old corridors and new infrastructural changes predicting probable movement paths of red deer.

A landscape fragment map was created with the same CORINE areas as the endemic habitat. Because of the frequently described preference of *D. reticulatus* for water proximity, the map was then refined to contain only those landscape fragments which were close to or contained a waterbody. Then, the variables temperature and rainfall were used for this map. Only those areas that contained the same or similar climatic conditions as the endemic habitat remained in the map. Altogether, six maps were compiled, expanding the minimum and maximum temperature range ± 20%, 15%, 10%, 5%, 2% and 0% of the total evaluated temperature range. A map resulting in a median number of potential sites was chosen for further analysis. In a next step, a map was compiled with PNV which belonged to the same large group as the vegetation in the endemic detection site. The landscape fragment map was overlaid with the PNV map and resulted in a map containing areas with an overlap between the two. Finally, this map was overlaid with red deer corridors in Bavaria. This final digital habitat map contained only areas including all parameters.

## 3. Results

### 3.1. Field Study on Dermacentor reticulatus

Ticks were collected from 2010 to 2013 during the spring and autumn activity periods of *D. reticulatus*. Altogether, 4085 ticks were collected at 60 sites in 17 sampling areas (Table 1; Supplementary Table S1). Among those were 3746 *Ixodes ricinus* (91.7%) and 339 *D. reticulatus* (8.3%). All *D. reticulatus* were adults, with 214 (63.1%) females and 125 (36.9%) males. *Dermacentor reticulatus* ticks were found at 12 out of 60 sampling sites (Table 1). Of the *I. ricinus*, 2095 were adults (1062 males and 1033 females), 1169 nymphs and 482 larvae. *Ixodes ricinus* were found at 47 sites, including all 12 sites which were positive for *D. reticulatus*. At 13 sampling sites, no ticks at all were found. All details on *I. ricinus* positive sites, as well as the negative sites, are shown in Supplementary Table S1.

The positive sites were located in 2 out of 3 sites in sampling area G7, in 9 out of 10 sampling sites in sampling area G3 and in 1 out of 5 sampling sites in area G1 (Figure 1, Table 1). *Dermacentor reticulatus* were found from April to May and late August until November. The area G3, located north of the city of Munich, was considered an endemic focus, as it was positive over several activity periods. All apart from one site in these areas were positive over at least two activity periods, whereas the other positive sites were positive only in one collection period each.

### 3.2. PCR Analysis for Babesia canis

Altogether, 308 questing *D. reticulatus* and 5 *D. reticulatus* ticks collected on hosts from these areas which were sent in to our laboratory from 2010–2012 were screened for *B. canis* DNA. Two samples were positive by PCR. Sequencing of one sample showed 100% similarity to *B. canis* isolate 5KO (Accession no. KF381412). The second sequence was not evaluable. Therefore, only one sample was considered positive. This sample was from the Isarauen (G3), i.e., 1 out of 301 samples from this area was positive, which results in an overall infection rate of 0.3% (95% CI: 0.01–1.84%).

### 3.3. Digital Habitat Characterisation

**Figure 2:**
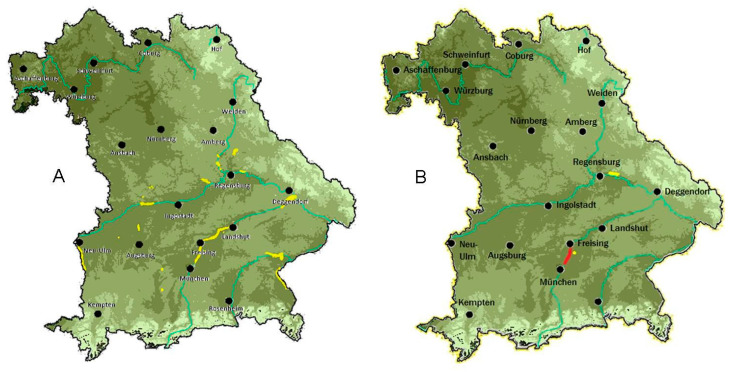
(**A**) Digital habitat map with 5% deviation; areas marked in yellow have suitable habitat conditions similar to the endemic habitat. (**B**) Localities where *Dermacentor reticulatus* and *Babesia canis* were found are shown in red.

The *D. reticulatus* ticks were found in three different habitat types: downy birch forest (Zengermoos, G1), hillside mixed forest with several small waterbodies (Regensburg, G7) and alluvial forests (river Isar, G3). The habitats are characterized by high humidity with marshy areas and soil wetness (Regensburg East, G7, and Zengermoos, G1). Due to the nearby international airport, the soil of the site north of Munich (G3) is characterized by changing soil wetness, because groundwater is pumped out regularly, followed by rising of the water level again. Red deer are present in the Isarauen (G3) and Zengermoos (G1). One of the digital red deer corridors coincides with the endemic habitat and is connected by two corridors to the positive site in Regensburg East (G7) and by one corridor to Zengermoos (G1); i.e., all habitats suitable for *D. reticulatus* and found positive were connected by red deer corridors. The first landscape fragment map revealed 1084 deciduous forests and 2380 mixed forest areas in Bavaria that included or were close to a waterbody. The map was refined according to temperature and rainfall. Working with a variation of 20% to the climate data of the endemic habitat, 1585 forest polygons remained; whereas with 0% variation, only 35 remained. Finally, 5% variation for rainfall and temperature was used with 345 remaining forest polygons. Overlay of this map with PNV and the red deer corridors revealed 15 forest areas with similar ecological characteristics to the endemic habitat, including the other two positively evaluated areas (Figure 2).

## 4. Discussion

The field study revealed *D. reticulatus* around the cities of Munich and Regensburg, which thus confirms previous reports [20,28,29]. None of the other investigated sites was positive for *D. reticulatus*. Several of the historic areas revisited in this study were taken from relatively old publications, and the landscape structure and use might have changed considerably, leading to different distributions of the tick. Comprehensive sampling of the entire area of Bavaria was not possible due to its size; therefore, sampling sites covered larger areas at random. Of course, this may bias the sampling success in a certain way, as it is known that *D. reticulatus* occurs highly focally distributed and in small local areas within larger sites [6]. This was confirmed both in studies on this tick species in Germany [10,28,29] and in other studies in other areas of Europe [30,31,32]. Adult *D. reticulatus* were found at a ratio of 1.7:1.0 females to males. We did not find any developmental stages of *D. reticulatus* during the field study. This can be explained because *D. reticulatus* larvae and nymphs are active mainly in the summer months from June to August when sampling in the present study was not performed [23,33] Furthermore, developmental stages of *D. reticulatus* show endophilic behavior, living in nests or deep in the vegetation [19], and are therefore usually found directly on their hosts, such as rodents and other small mammals [12,13]. This study confirmed a preference of *D. reticulatus* for humid natural deciduous and mixed alluvial forests along rivers, swamps and moors [6,11]. However, recent findings showed *D. reticulatus* also along rivers without alluvial forests, e.g., in studies in Poland and Slovakia [34,35]. (Rubel et al. (2016) [36] published an updated overview of the distribution of *D. reticulatus* in Europe based on published literature using georeferenced positions. In this study, they defined the distribution range as 41–57° N latitude. For Bavaria, almost 8000 mixed forest areas are close to waterbodies, suggesting that Bavaria contains several more suitable habitats for *D. reticulatus*. This tick species could not be found in the present study in habitats with a drier character, even though one *D. reticulatus* was sent in to our laboratory from the Main valley (Würzburg), which is one of the warmest and driest areas in Germany, and *D. reticulatus* has also been described in other dry areas in Europe only recently [37,38]. The activity of *D. reticulatus* was within the temperature range described previously [39]. The digital habitat analysis was interesting concerning the occurrence of red deer, with the red deer population in the endemic area Isarauen (G3) exactly covering the extent of the *D. reticulatus* positive area.

Two habitats positive for *D. reticulatus* were also known habitats for red deer, further supporting the role of red deer in the dispersal of *D. reticulatus*. This is further supported by results of a previous study, where significantly more *D. reticulatus* were found on red deer than on roe deer [3].

The total prevalence for *B. canis* in the investigated *D. reticulatus* population in the endemic habitat was similarly low as in questing ticks in Saarland [8]. Other direct detections of *B. canis* in questing *D. reticulatus* have been described previously with variable prevalence, for example, in Hungary (8.2%) [40], Serbia (20.8%) [41], Slovakia (1.8–14.7%) [42,43] and the Netherlands (1.64%) [44]. In Switzerland, 19 out of 23 questing *D. reticulatus* in an outbreak area of canine babesiosis were positive [45]. On the other hand, questing *D. reticulatus* in Poland were negative for *B. canis* DNA, even though canine babesiosis occurs in the area [46].

## 5. Conclusions

The digital habitat characterization revealed a further 15 forest areas in Bavaria with the same ecological conditions as the endemic area. As the map showed a positive prognostic value, it should be considered in further targeted field investigations in the area.

Isarauen (G3) is a recreational area highly frequented also by dog owners and their dogs, showing the potential for attracting canine babesiosis in this area in southern Germany.

The detection of *B. canis* in questing *D. reticulatus* in the area of Isarauen (G3) undoubtedly requires preventive measures for dogs in this highly frequented recreational *D. reticulatus*-endemic area.

## Figures and Tables

**Table 1:** Georeferenced sampling results for *Dermacentor reticulatus* in Bavaria from 2010 to 2013 *.

								*Dermacentor reticulatus* (*n*)		
Area	Site	Geographic Coordinates	Altitude	Temperature at 50 cm (°C)	Soil Temperature at 5 cm Depth (°C)	Relative Humidity (%)	General Weather Impression	M	F	Total
G1—Munich	Zengermoos	48°17′21.39″ N/11°46′1.19″ E	472	19.7	22	56	sunny	1	0	1
G3—Lower Isarauen (Munich to Freising)	Garching to Mintraching	48°14′36.82″ N/11°40′18.42″ E	488	16.2	16	40.5	overcast	0	10	10
	Pulling to Mintraching	48°21′42.82″ N/11°43′12.02″ E	447	23	20	55	overcast	1	2	3
	Achering to Garching North	48°22′6.42″ N/11°43′55.69″ E	455	20.3	20	59	sunny after previous rain	7	9	16
	Ismaning to Fischerhäuser	48°15′3.69″ N/11°41′3.82″ E	483	16.1	17	64	overcast	0	10	10
	Fischerhäuser	48°16′7.39″ N /11°41′53.55″ E	477	22	19	44.5	sunny	4	7	11
	Airport	48°18′44.34″ N/11°42′8.74″ E	459 **	25.1 23.4	21 25	65.9 54.7	sunny sunny	131	172	33
	Hallbergmoos	48°18′44.34″ N/11°42′8.74″ E	465	19.4	19	42.7	windy	0	3	3
	Pförrerau	48°22′49.79″ N/11°44′37.30″ E	437	19.6	18	58.5	sunny	0	1	1
	Zwillingshof to Fischerhäuser	48°16′56.82″ N/11°42′11.52″ E	481 ***	20	19	52.8	rainy	27	45	245
				19.3	20.5	53.6	sunny	18	30	
				11.5	17	49.5	not recorded	10	20	
				20	19	52.8	overcast	25	42	
				23.1	21	58	sunny	16	12	
G7—Regensburg East	Bach a.d. Donau	49°1′55.46″ N/12°17′36.00″ E	406	25.2	16	55.2	sunny	0	1	1
	Frauenzell Wiesent	49°2′20.98″ N/12°22′19.96″ E	352	26.2	16	50	sunny	2	3	5
Total								125	214	339

* Negative sites are not shown. ** Two collections were carried out on 09.09.2011 and 22.08.2013. *** Five collections were carried out on 07.09.2010, 23.09.2010, 03.11.2011, 27.10.2011 and 23.08.2013.

## Supplementary Materials

The following are available online at https://www.mdpi.com/2076-0817/9/7/541/s1, Table S1: Georeferenced sampling results for *Dermacentor reticulatus* and *Ixodes ricinus* in Bavaria from 2010 to 2013 at 60 sites within 17 sampling areas.
