# Supplementary material for: Dermacentor reticulatus and Babesia canis in Bavaria (Germany)—A Georeferenced Field Study with Digital Habitat Characterization
Source: Pathogens. 2020 Jul 7;9(7):541. doi: 10.3390/pathogens9070541 (PMC7400213; doi:10.3390/pathogens9070541)
Supplement: Supplementary file 1 [file pathogens-09-00541-s001.pdf]

Supplementary Materials

Supplementary Table 1: Georeferenced sampling results for *Dermacentor reticulatus* and *Ixodes ricinus* in Bavaria from 2010 to 2013 at 60 sites within 17 sampling areas.

				Ixodes ricinus					*Dermacentor reticulatus*		
**Area**	**Site**	**Geographic coordinates**	**Altitude**	**M**	**F**	**L**	**N**	**Total**	**M**	**F**	**Total**
G1—Munich	Daglfing	48°8' 18.81"N/11°39' 18.42"E	514	0	0	0	0	0	0	0	0
	Mallertshofer Holz	48°16'11.26"N/11°36'37.83"E	514	4	0	0	3	7	0	0	0
	Inhausen	48°17' 43.08"N/11°33' 18.45"E	473	5	2	0	1	8	0	0	0
	Schwarzhölzl	48°14' 34.89"N/11°29' 47.87"E	486	15	23	252	117	407	0	0	0
	Zengermoos	48°17' 21.39"N/11°46' 1.19"E	472	93	74	169	1	337	1	0	1
G2—Isarauen* South of Munich	Straßlach	48°0' 31.43"N 11°29' 45.94"E	635	3	2	0	0	5	0	0	0
G3—Lower Isarauen (München to Freising)	Dietersheim	48°16' 49.73"N/11°41' 18.92"E	464	0	0	0	0	0	0	0	0
	Garching to Mintraching	48°14'36.82"N/11°40'18.42"E	488	27	31	0	33	91	0	10	10
	Pulling toMintraching	48°21' 42.82"N/11°43' 12.02"E	447	3	5	0	0	8	1	2	3
	Achering to Garching North	48°22'6.42"N/11°43'55.69"E	455	1	2	0	0	3	7	9	16
	Ismaning toFischerhäuser	48°15'3.69"N/11°41'3.82"E	483	6	5	0	1	12	0	10	10
	Fischerhäuser	48°16'7.39"N /11°41'53.55"E	477	0	1	0	0	1	4	7	11
	Airport	48°18'44.34"N/ 11°42'8.74"E	459	1	0	0	0	1	14	19	33
	Hallbergmoos	48°18'44.34"N/11°42'8.74"E	465	0	1	0	0	1	0	3	3
	Pförrerau	48°22' 49.79"N/11°44' 37.30"E	437	1	0	0	0	1	0	1	1

	Zwillingshof to Fischerhäuser	48°16'56.82"N/11°42'11.52"E	481	23	21	0	4	48	96	149	245
G4—Isarauen between Freising and Landshut	Lower Schwabenau	48 °24' 0.27"N/11 °45' 40.64"E	443	18	18	0	11	47	0	0	0
	Marzling	48 °24' 6.38"N/11 °47' 34.74"E	440	78	74	0	22	174	0	0	0
	Gaden	48 °24' 30.12"N/11 °51' 47.88"E	433	1	3	0	0	4	0	0	0
	Niederhummel	48 °25' 54.09"N/11 °53' 19.42"E	425	43	46	0	17	106	0	0	0
	Grünseiboldsdorf	48 °26' 18.38"N/11 °54' 18.59"E	420	4	2	0	1	7	0	0	0
	Edlkofen	48 °30' 27.89"N/11 °59' 29.97"E	403	0	0	0	0	0	0	0	0
	Niederaichbach	48 °36' 13.02"N/12 °19' 39.98"E	410	5	11	0	0	16	0	0	0
G5—Estuary of the river Isar	Estuary 1	48 °48' 3.44"N/12 °56' 16.83"E	319	25	25	0	77	127	0	0	0
	Niederpöring	48 °43' 5.03"N/12 °50' 13.53"E	325	112	95	20	162	389	0	0	0
	Pielweichs	48 °45' 13.72"N/12 °51' 0.03"E	319	28	27	30	47	132	0	0	0
	Estuary 2	48 °59' 25.63"N/12 °0' 41.89"E	382	0	2	0	1	3	0	0	0
	Forstern	48 °46' 43.49"N/12 °54' 54.63"E	319	1	2	0	0	3	0	0	0
	Plattling	48 °47' 55.86"N/12 °57' 16.61"E	309	59	55	0	17	131	0	0	0
G6—Regensburg	Tegernheim	49 °1' 3.49"N/ 12 °9' 56.13"E	319	0	0	0	0	0	0	0	0
	Sinzing	48 °59' 25.63"N/12 °0' 41.89"E	382	56	38	0	107	201	0	0	0
	Aberdeenpark	49°2'41.85"N/12°6'19.30"E	389	0	0	0	0	0	0	0	0
G7—Regensburg East	Pillnach	48 °58' 38.08"N/12 °30' 23.92"E	410	1	3	0	0	4	0	0	0
	Bach a.d. Donau	49 °1' 55.46"N/12 °17' 36.00"E	406	2	1	0	4	7	0	1	1
	Frauenzell Wiesent	49 °2' 20.98"N/12 °22' 19.96"E	352	7	8	0	6	21	2	3	5
G8—Ingolstadt	Bergheim	48 °45' 6.80"N/11 °17' 15.79"E	375	0	0	0	0	0	0	0	0
	Bittenbrunn	48 °45' 19.63"N/11 °8' 54.84"E	449	56	53	0	40	149	0	0	0
G9—Ichenhausen	Ettenbeuren	48 °22' 30.45"N/10 °22' 42.03"E	513	30	6	1	10	47	0	0	0

G10—Western Forest South, "Stauden"	Grimoldsried	48°13' 13.07"N/10°37' 13.54"E	587	23	24	0	58	105	0	0	0
	Langenneufnach	48°14' 49.09"N/10°37' 1.18"E	581	3	4	0	68	75	0	0	0
	Elmischwang	48°16' 54.76"N/10°37' 35.79"E	516	33	52	0	100	185	0	0	0
G11—Zusmarshausen South	Dinkelscherben	48°21' 28.45"N/10°35' 34.50"E	517	20	22	0	17	59	0	0	0
	Rommelsried	48°22' 10.02"N/10°42' 52.43"	507	17	27	0	46	90	0	0	0
	Rothsee	48°23' 39.65"N/10°37' 3.35"E	446	14	20	0	1	35	0	0	0
G12—Western Forest North, "Holzwinkel"	Wörleschwang	48°25' 34.41"N/10°37' 5.58"E	497	46	38	0	101	185	0	0	0
	Emersacker	48°29' 4.41"N/10°41' 17.49"E	510	96	96	0	23	215	0	0	0
	Welden	48°27' 15.47"N/10°41' 0.96"E	495	92	94	0	37	223	0	0	0
	Horgau	48°24' 22.73"N/10°42' 27.48"E	494	3	4	0	0	7	0	0	0
G13—Lechauen**	Langweid a. Lech	48°29' 48.53"N/10°52' 27.77"E	427	0	0	0	0	0	0	0	0
	Oberottmarshausen	48°14' 17.76"N/10°55' 1.10"E	512	0	0	0	0	0	0	0	0
G13—Würzburg	Veitshöchheim	49°50' 43.44"N/9°53' 37.47"E	256	1	7	0	1	9	0	0	0
	Margetshöchheim	49°49' 35.28"N/9°51' 7.53"E	252	2	4	0	4	10	0	0	0
	Königswäldchen	49°48' 34.17"N/9°53' 42.88"E	246	0	0	0	0	0	0	0	0
	Sieboldspark	49°46' 49.26"N/9°57' 29.76"E	263	0	0	0	0	0	0	0	0
	Gerbrunn	49°45' 41.26"N/9°59' 44.63"E	282	0	0	0	0	0	0	0	0
G 15—Mühlhausen	Weihersdorf	49°10' 38.28"N/11°28' 21.66"E	473	2	2	0	4	8	0	0	0
G16—Nürnberg	Pegnitz	49°42' 19.55"N/11°33' 16.29"E	388	1	3	10	26	40	0	0	0
	Dehnberg (Lauf a. d. Pegnitz)	49°32' 20.79"N/11°17' 41.36"E	354	1	0	0	1	2	0	0	0
G17—Michelau	Kemmern	49°56' 28.20"N/10°51' 55.48"E	238	0	0	0	0	0	0	0	0
	Breitengüßbach	49°58' 45.21"N/10°52' 46.69"E	245	0	0	0	0	0	0	0	0
Total				1062	1033	482	1169	3746	125	214	339

*Meadows along the river Isar; ** meadows along the river Lech.
